# Retrospective evaluation of two-year results with a filtering trabeculotomy in comparison to conventional trabeculectomy by exact matching

**DOI:** 10.12688/f1000research.26772.2

**Published:** 2021-06-04

**Authors:** Alicja Strzalkowska, Peter Strzalkowski, Yousef Al Yousef, Jost Hillenkamp, Franz Grehn, Nils A. Loewen

**Affiliations:** 1 Department of Ophthalmology, University of Würzburg, Würzburg, Bavaria, 97080, Germany

**Keywords:** trabeculectomy, mitomycin C, trabeculotomy, exact matching, open-angle glaucoma, iridectomy

## Abstract

**Background:** To compare two-year results of a filtering trabeculotomy (FTO) to conventional trabeculectomy (TE) in open-angle glaucoma by exact matching.

**Methods:** 110 patients received an FTO and 86 a TE. FTO avoided the need for an iridectomy due to a preserved trabeculo-descemet window anterior to the scleral flap. TE employed a trabecular block excision and iridectomy. Mitomycin C was used in both. FTO and TE were exact matched by baseline intraocular pressure (IOP) and the number of glaucoma medications. Complete and qualified success (IOP ≤18 mmHg and IOP reduction ≥ 30%, with or without medication) were primary endpoints. IOP, visual acuity (BCVA), complications and intervention were secondary endpoints.

**Results:** 44 FTO were exact matched to 44 TE. The IOP baseline in both groups was 22.5±4.7 mmHg on 3±0.9 medications. At 24 months, complete success was reached by 59% in FTO and 66% in TE, and qualified success by 59% in FTO and 71% in TE. In FTO, IOP was reduced to 12.4±4.3 mmHg at 12 months and 13.1±4.1 mmHg at 24 months. In TE, IOP was 11.3±2.2 mmHg at 12 months and 12.0±3.5 mmHg at 24 months. Medications could be reduced at 24 months to 0.6±1.3 in FTO and 0.2±0.5 in TE. There were no significant differences between the two groups in IOP, medications, complications or interventions at any point.

**Conclusion: **Modifying aqueous flow through a limited trabeculotomy in FTO yielded clinical outcomes similar to traditional TE but allowed to avoid an iridectomy.

## Introduction

Since the first description of a guarded filtering procedure in enucleated eyes by Grant in 1958 ^1^, performance of this surgery in patients in 1961 by Sugar ^2^, popularization of the term “trabeculectomy” in 1968 by Cairns ^3^, and introduction of mitomycin-C as an antifibrotic to make it more effective ^4^, trabeculectomy (TE) has remained a primary surgery in the treatment of glaucoma ^5^. Over the years, multiple modifications of this surgery have been explored to improve its effectiveness, to make outcomes more predictable and to reduce postoperative complications and need for interventions. These modifications include, among others, variations in size, localization, and thickness of the scleral-flap, different suture techniques, variable intra- and postoperative treatment with antifibrotics or a combination of these approaches with one another ^6^.

In this study, we combined elements of deep sclerectomy ^7, 8^ and trabeculotomy ^9^ with TE in an attempt to improve conventional outflow as well as subconjunctival aqueous humor drainage. Encouraged by a pilot study of filtering trabeculotomy (FTO) with a complete success rate of 79% ^10^, we hypothesized that FTO had a higher success rate and lower complication rate than TE. We applied advanced statistics, *exact matching* ^11, 12^, to enable a highly balanced comparison of our retrospective data with two-year follow-up.

## Methods

### Study design

This retrospective study was approved by the ethics committee of the University of Würzburg, Germany (#2019101601AS). Because of its retrospective nature, informed consent was waived.

All primary open angle glaucoma (POAG) patients at the University Eye Hospital Würzburg who underwent a modified FTO with mitomycin C (MMC) or TE with MMC by a single surgeon (FG) between 2007 and 2014 were analyzed. The indication for surgery was a failure to control intraocular (IOP) despite maximally tolerated medical therapy. Only one eye was included per patient. If two eyes had been operated on, the first eye was chosen to take advantage of a longer history.

Patients were matched by baseline IOP and the number of glaucoma medications.

From patient records, we obtained from 2019 to 2020 the medical history, the best-corrected visual acuity assessed (BCVA [logMAR]), IOP (Goldmann tonometry [mmHg]), topical glaucoma medications (including a glaucoma medication score (GMS) ^13^), as well as postoperative events and complications. These included hypotony with choroidals, hypotony maculopathy, flat or shallow chamber, an IOP relatively high for surgical glaucoma (above 25 mmHg), bleb leakage, a persistently flat bleb indicating absent flow, hyphema, iris incarceration and need for cataract surgery. We computed the complete and qualified success, according to the guidelines set forth by the World Glaucoma Association ^14^. In patients who had either TE or FTO in both eyes, the first eye was chosen to be included in the study. Complete success was defined as a postoperative IOP of ≤18 mmHg with a reduction of ≥30% from baseline without glaucoma medications. Qualified success was a postoperative IOP of ≤18 mmHg with a reduction of ≥30% from the baseline, achieved with or without glaucoma medications ^14^. Follow-up visits occurred 7 days, 1 month, 3 months, 6 months, 12 months, and 24 months after surgery.

### Exclusion criteria

The exclusion criteria consisted of patients aged below 18 years, glaucoma types other than open-angle glaucomas, and a history of TE, trabeculotomy or glaucoma drainage device implantation.

### Surgical technique

The eye was rotated downward with a traction suture. A 5 mm fornix-based peritomy was made at the anatomic 12 o’clock position, and a sub-tenon pocket was fashioned to accommodate a sponge soaked with MMC at a concentration of 0.2 mg/ml and 100 µl volume for 3 minutes.

In TE, a 3 mm × 4 mm half scleral thickness flap was created. A 0.8 mm × 2 mm sclerotrabecular block was excised to enter the anterior chamber, as described before ^10^. A peripheral iridectomy was made. The scleral flap was secured with 10.0 nylon to allow visible percolation of aqueous, and the conjunctiva was closed with an interlocking running suture resulting in a diffusely forming bleb ^15^.

In FTO, the scleral flap was sized 4 mm × 4 mm. A smaller, tongue-shaped flap was dissected underneath to unroof and create access ostia to Schlemm’s canal. This second flap was excised similar to deep sclerectomy. The canal was probed with a metal trabeculotomy probe (Mackensen, Geuder Inc., Heidelberg) on both sides while the trabeculo-descemet window at the base of the scleral flap was preserved so that no bulk aqueous outflow could occur and no iridectomy was needed. The remaining steps were identical to those in TE.

Postoperative drops consisted of dexamethasone six times for the first week, which was tapered by one drop per week. Ciprofloxacin eye drops were applied four times a day for one week. All patients received 5 mg of 5-FU (0.1 cc volume) daily for a week unless IOP was below 5 mmHg or a Seidel-positive bleb leak or a corneal erosion was present. 5-FU was also given once a week for the first month during return visits using the same criteria.

### Statistics

Statistical analyses were performed using Statistica 13.1 (StatSoft, Tulsa, Oklahoma, United States) and MedCalc (MedCalc 19.1.3, Ostend, Belgium). A total of 88 patients (1:1, FTO:TE) were matched with exact matching ^11, 12^ based on the baseline IOP and glaucoma medications.

Categorical variables were described as the frequency with percentage, whereas continuous and discrete variables as mean with standard deviation (SD) or median with range. The chi-squared test or Fisher’s exact test was used for the analysis of categorical variables. Continuous variables were compared using Student’s t-test or Mann–Whitney U test, whereas discrete variables were compared using Mann-Whitney U test. The distribution of continuous variables was determined by the Shapiro-Wilk test, equality of variances by Levene’s test. Assessment of repeated measures for IOP was performed using repeated measures MANOVA and Tukey’s test, while visual acuity (logMAR) and medications were examined using the Friedman test and Wilcoxon signed-rank test. The success of treatment was expressed by a Kaplan-Meier curve and compared between treated groups using the log-rank test. The success of treatment at particular a given time point was determined by odds ratio (OR) with respective 95% confidence intervals and p-values. P-values below 0.05 were considered statistically significant.

Our power calculation before commencing the study indicated that we would need 39 eyes per group to detect a difference of at least 20% at a power of 80% and alpha of 0.05 (continuous endpoints, two independent sample study). We then performed a post-hoc analysis for IOP at year 1 and 2 and determined the effect size using Cohen’s d (d (.01) = very small, d (.2) = small, d (.5) = medium, d (.8) = large, d (1.2) = very large, and d (2.0) = huge.

## Results

A total of 196 patients were included. The unmatched demographic data of FTO and TE had significant differences in preoperative IOP (p=0.017), glaucoma medication score (p=0.001), and pseudophakia (p=0.012). In total 88, eyes (44 in each group) could be matched as exact pairs eliminating key differences in IOP and medications. The IOP at baseline was 22.6±4.7 mmHg in FTO and 22.6±4.7 in TE while on 3.0±0.9 medications in both ( Table 1). There were no significant differences between FTO and TE in gender, age, best-corrected visual acuity, type of glaucoma, or surgical side. In FTO, 13 eyes were pseudophakic compared to 3 in TE (p=0.006). Two patients in FTO had a pars plana vitrectomy and one a retinal cryopexy. In TE, there were no prior ocular surgeries other than phacoemulsification.

**Table 1.** Demographic data of both groups after matching.

	FTO n=44	TE n=44	p-value
Female (n, (%))	18 (41)	21 (48)	0.520
Male (n, (%))	26 (59)	23 (52)	
Age, years (mean±SD)	65±13	68±9	0.483
BCVA, logMAR (mean±SD)	0.2±0.3	0.2±0.3	0.923
Preoperative IOP, mmHg (mean±SD)	22.5±4.7	22.5±4.7	0.891
Glaucoma medication (mean±SD)	3±0.9	3±0.9	0.755
Type of glaucoma (n, (%))			0.087
POAG	33 (75)	33 (75)	
PXG	7 (16)	11 (25)	
PG	4 (9)	0 (0)	
Right	21 (48)	26 (59)	0.285
Left	23 (52)	18 (41)	
Pseudophakia (n, (%))	13 (30)	3 (7)	0.006
Prior ocular surgery excluding phaco (n, (%))	3 (7)	0 (0)	0.116
Type of prior laser (n, (%))			
ALT	13 (30)	12 (27)	0.813
SLT	3 (7)	0 (0)	0.241
CPC	4 (9)	3 (7)	1.000
nd:YAG capsulotomy	1 (2)	0 (0)	1.000

FTO: filtering trabeculotomy; TE: trabeculectomy; BCVA: best-corrected visual acuity assessed; IOP: intraocular pressure; POAG: primary open angle glaucoma; PXG: pseudoexfoliation glaucoma; PG: pigmentary glaucoma; ALT: argon laser trabeculoplasty; SLT: selective laser trabeculoplasty; CPC: cyclophotocoagulatoin; nd:YAG: neodymium-doped yttrium aluminum garnet

**Figure 1.**
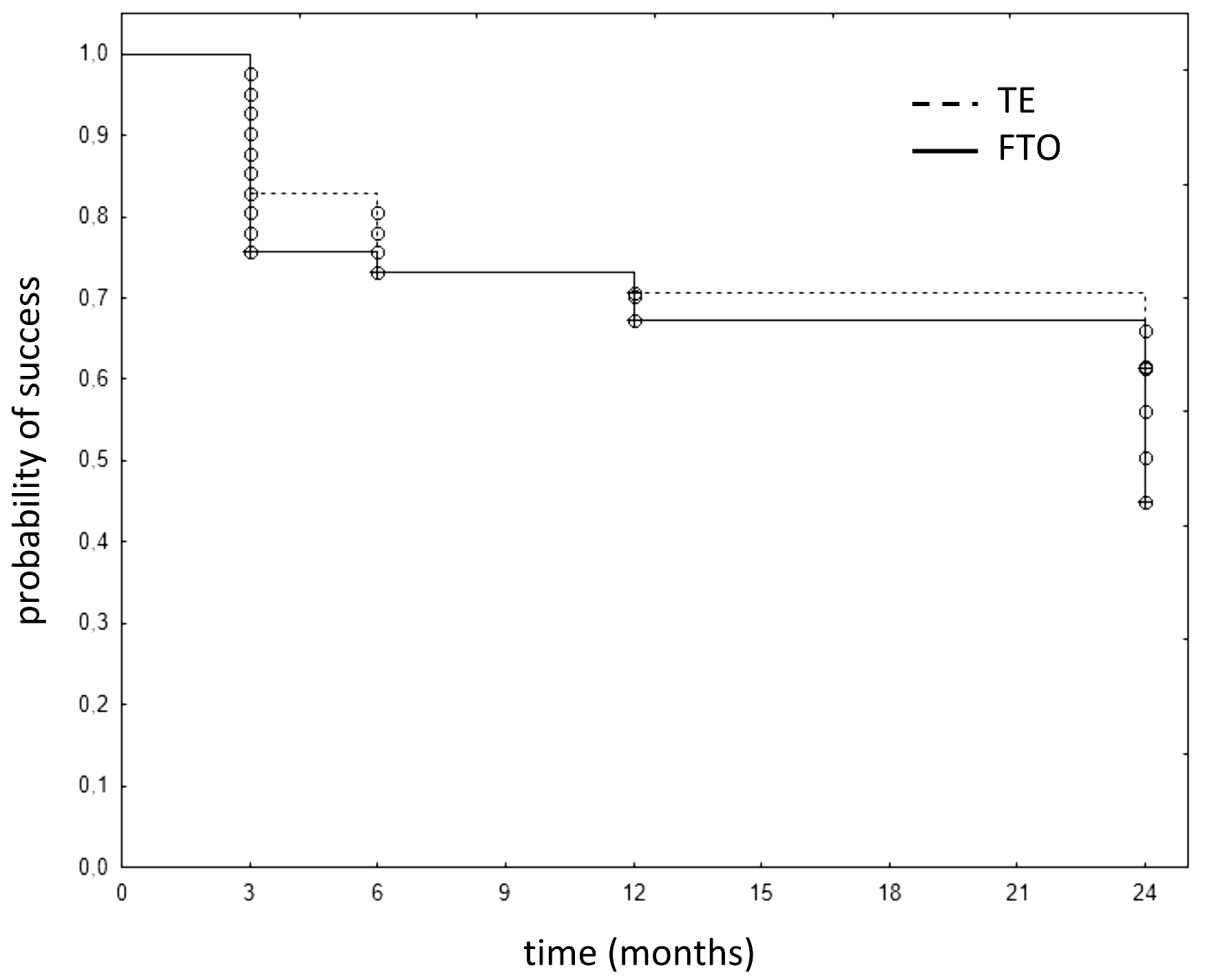
Kaplan-Meier curve for complete success in filtering trabeculotomy (FTO) and trabeculectomy (TE).

**Figure 2.**
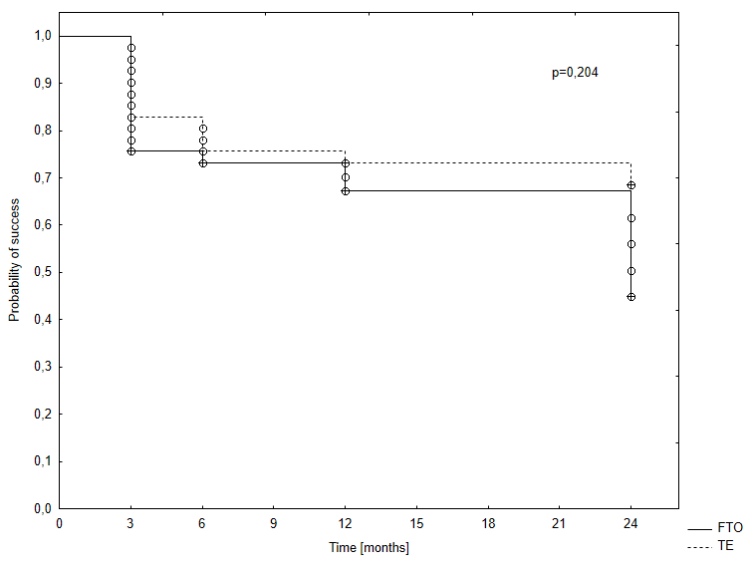
Kaplan-Meier curve for qualified success in FTO and TE.

**Figure 3.**
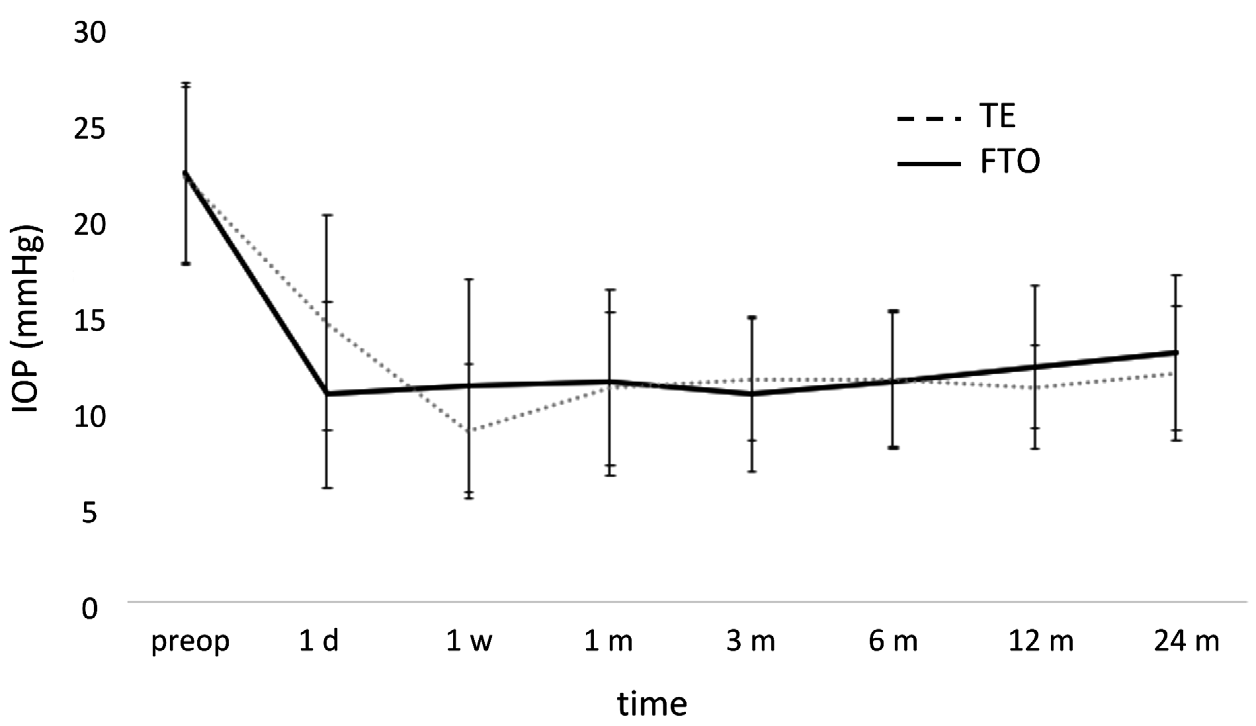
Mean preoperative vs postoperative intraocular pressure (IOP) of filtering trabeculotomy (FTO) and trabeculectomy (TE) (mean±SD).

**Figure 4.**
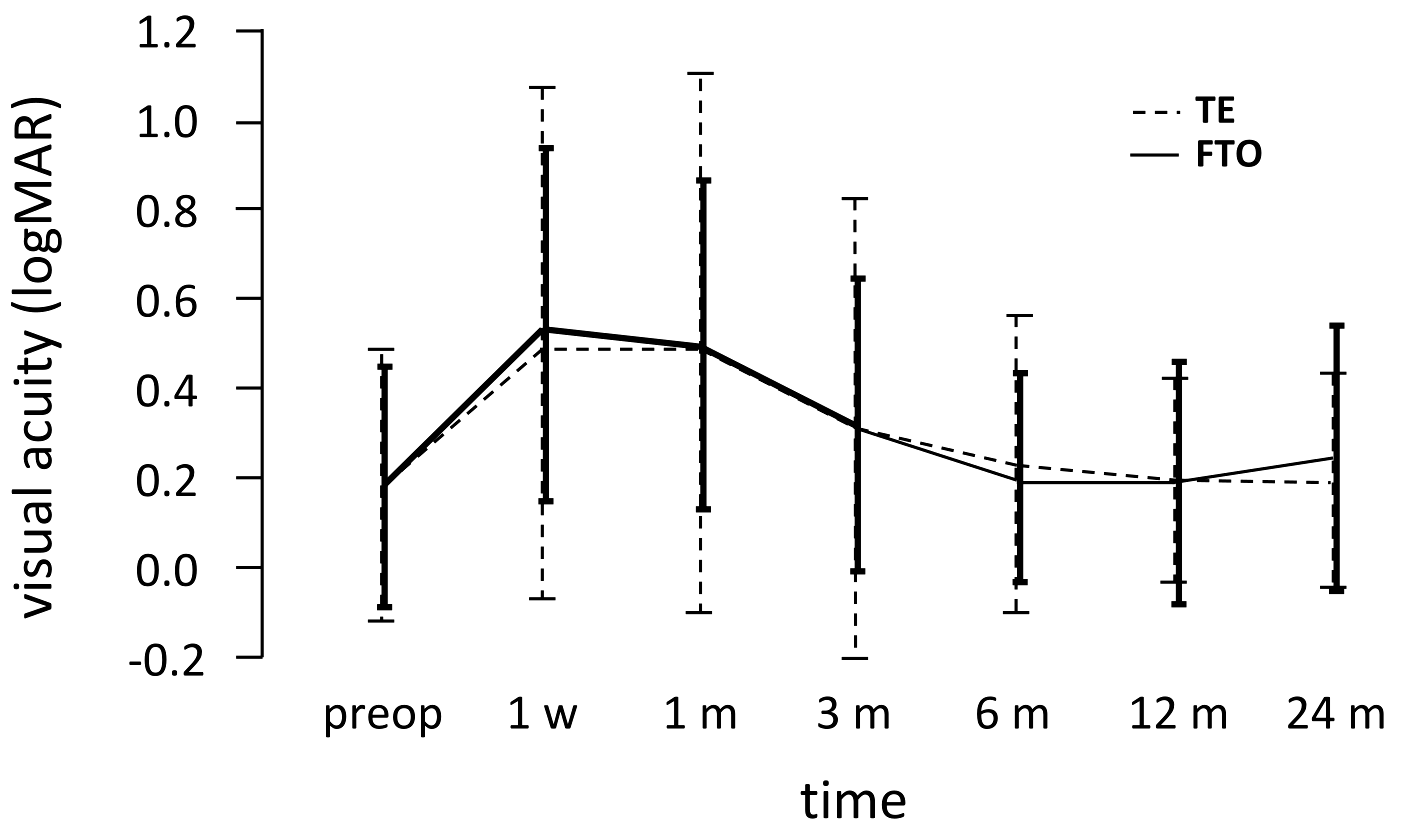
Preoperative versus postoperative visual acuity of both groups (mean±SD). FTO: filtering trabeculotomy; TE: trabeculectomy.

**Figure 5.**
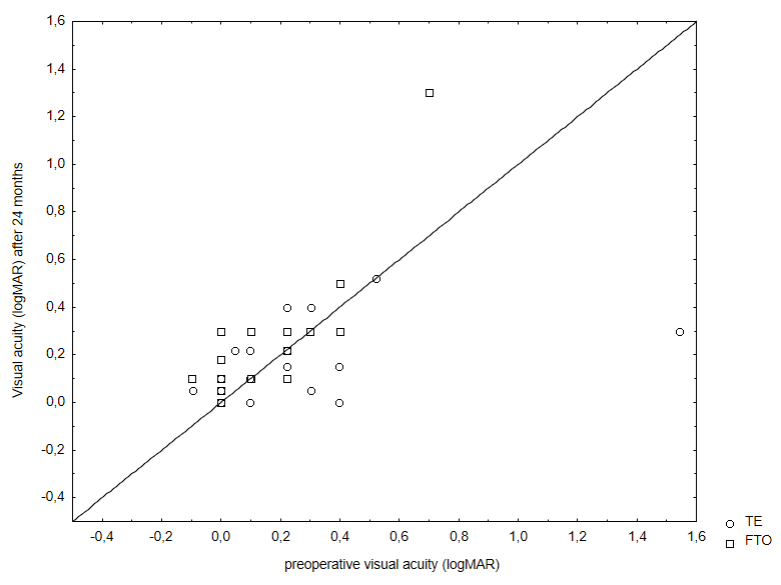
Scatter plots of visual acuity of both groups.

There were no statistically significant inter-group differences in complete or qualified success at any time (p=0.403 for complete success; p=0.204 for qualified success at 24 months; Figure 1 and Figure 2). The complete success rate in FTO ranged from 79% at 6 to 78% at 12 months, and 59% at 24 months. In TE, it was 81%, 85%, and 66%, respectively. Similarly, IOPs of FTO and TE were not significantly different at any time (12 months: p=0.983, 24 months: p=1.000, Figure 3). At one year, the IOP had declined to 12.4±4.3 mmHg in FTO and 11.3±2.2 mmHg in TE with medications (qualified success). At two years, IOP remained at 13.1±4.1 mmHg in FTO and 12.0±3.5 mmHg in TE (qualified success), respectively. The posthoc analysis showed that we could have detected an IOP difference of 16.1% at one year and an IOP difference of 17.6% at two years with a power above 80%. Cohen’s d was 0.32 at one year and 0.29 at two years, respectively, in both cases classifying as small. The postoperative visual acuity was not significantly different in FTO and TE at any time (p=0.894 after 12 months; p=0.443 after 24 months; Figure 4 and Figure 5).

**Figure 6.**
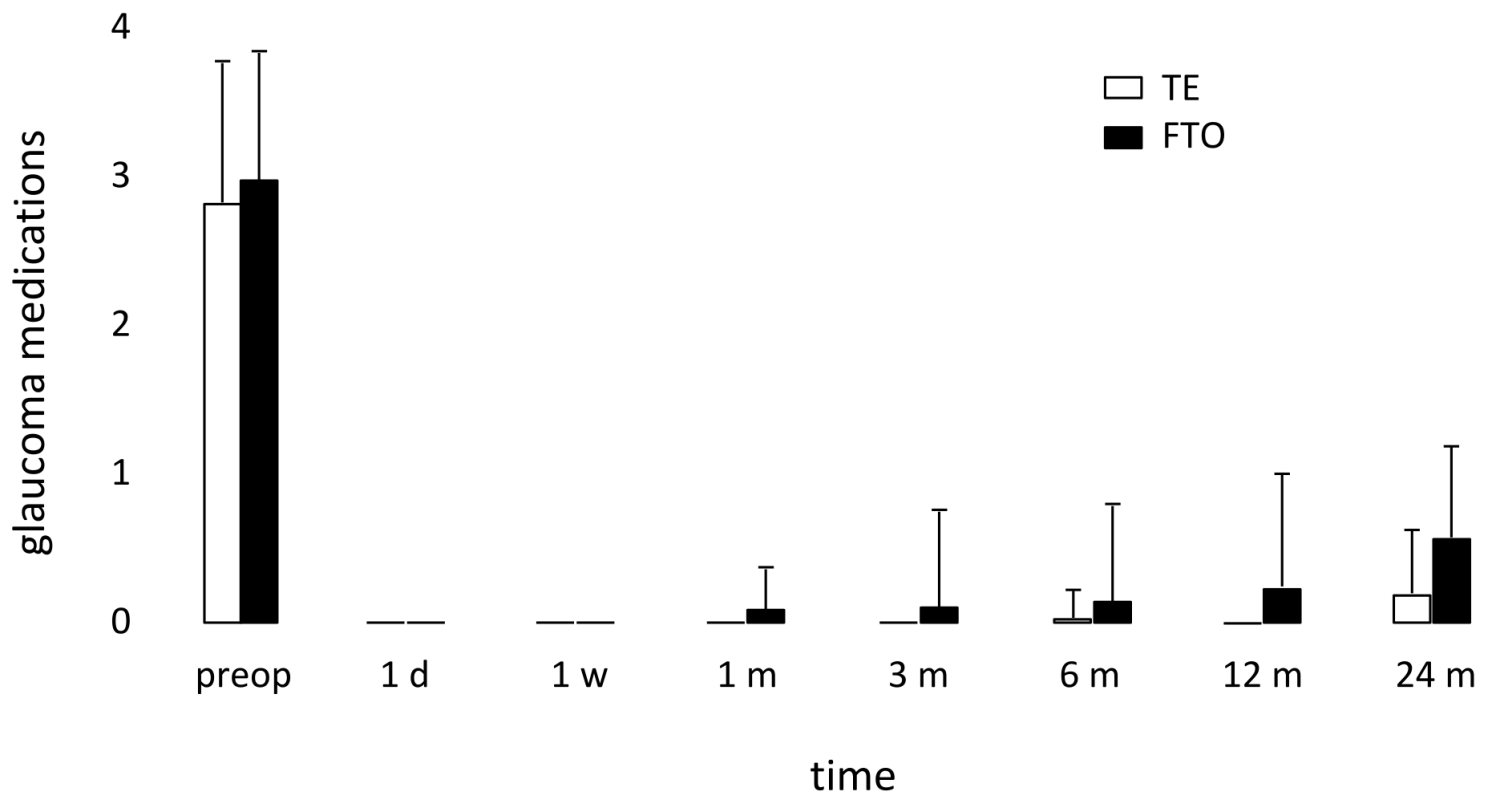
Preoperative versus postoperative glaucoma medications of both groups (mean±SD). FTO: filtering trabeculotomy; TE: trabeculectomy.

**Figure 7.**
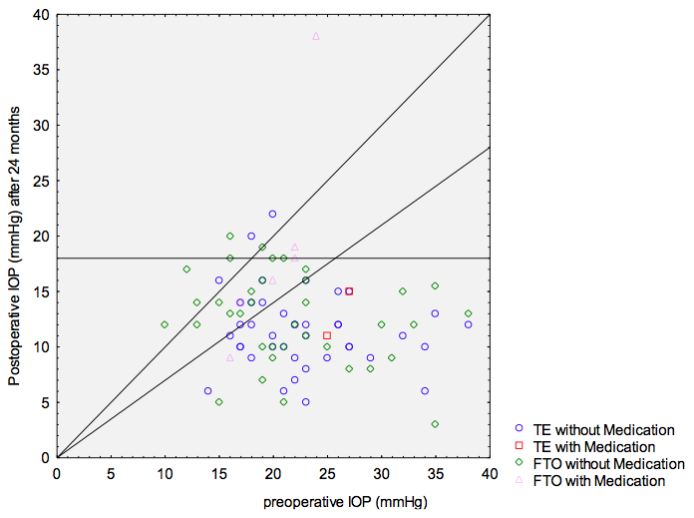
Scatter plots of IOP of both groups. Few patients required medications postoperatively.

There was a reduction in glaucoma medication from 3±0.9 to 0.6±1.3 in FTO and from 3±0.9 to 0.2±0.5 in TE after 24 months. There was no significant difference between the two groups in glaucoma medications at 24 months with 19% of patients in FTO and 12% of patients in TE using glaucoma drops (p=0.471, Figure 6 and Figure 7).

Postoperative complications in FTO included 5 eyes (12%) with a high IOP, hyphema in 5 cases (11%) and hypotony (IOP ≤ 5 mmHg ^16^) with choroidals in 4 eyes (9%). The most common postoperative challenge in TE was a flat bleb in 6 eyes (14%), hyphema in 3 eyes (7%), high IOP in 2 eyes (5%), bleb leakage in 2 eyes (5%), and hypotony in 1 eye (2%). A total of 16, mostly reversible, complications occurred in each group (37%; Table 2).

**Table 2.** Postoperative complications and challenges.

	FTO, n (%)	TE, n (%)	p-value
Hypotony with choroidals	4 (9)	1 (2)	0.360
Hypotony maculopathy	0 (0)	0 (0)	1.000
Shallow or flat chamber	4 (9)	4 (9)	1.000
High IOP	5 (12)	2 (5)	0.266
Bleb leakage	0 (0)	2 (5)	0.494
Flat bleb	1 (2)	6 (14)	0.110
Hyphema	5 (12)	3 (7)	0.713
Iris incarceration	0 (0)	1 (2)	1.000
Need for cataract surgery	1 (1)	1 (1)	1.000

FTO: filtering trabeculotomy; TE: trabeculectomy; IOP: intraocular pressure.

The number of postoperative interventions was the same in the two groups (p=0.087). Bleb needling, conjunctival suture, and scleral flap suture were the most common interventions. There was no statistically significant difference between both groups in early or late interventions ( Table 3), except 5-FU and laser suture lysis, which was performed more often in FTO.

**Table 3.** Postoperative interventions in FTO and TE.

Early postoperative interventions	FTO (n, (%))	TE (n, (%))	P-Value
No. of interventions 0 1 2 3 4	26 (59) 15 (34) 2 (5) 1 (2) 0 (0)	35 (80) 8 (18) 0 (0) 0 (0) 1 (2)	0.087
5-FU (mean±SD)	2.1±2.4	0.8±0.9	0.023
Bleb needling	11 (25)	6 (14)	0.280
Conjunctival suture	4 (9)	3 (7)	1.000
Scleral flap revision (high IOP)	2 (5)	0 (0)	0.494
Scleral flap suture (hypotony)	2 (5)	2 (5)	1.000
nd:YAG laser goniopuncture	1 (2)	0 (0)	1.000
Cyclodestruction	1 (2)	0 (0)	1.000
Iris repositioning	1 (2)	1 (2)	1.000
Laser suture lysis (average±SD)	1.5±1.5	0.8±0.9	0.026
Late postoperative interventions			
No. of interventions 0 1 2	35 (80) 6 (14) 3 (7)	42 (95) 2 (5) 0 (0)	0.147
Bleb needling	1 (2)	2 (5)	1.000
Conjunctival suture	3 (7)	0 (0)	0.241
Scleral flap revision (high IOP)	3 (7)	0 (0)	0.241
Scleral flap suture (hypotony)	0 (0)	0 (0)	**
nd:YAG laser goniopuncture	4 (9)	0 (0)	0.116
Cyclodestruction	1 (2)	0 (0)	1.000
Re-TE	0 (0)	0 (0)	**

** not calculated. FTO: filtering trabeculotomy; TE: trabeculectomy; IOP: intraocular pressure

## Discussion

TE with MMC remains a primary surgery in the management of advanced glaucoma ^5^ despite its potential for serious complications that include choroidal effusions, maculopathy, blebitis, endophthalmitis, and suprachoroidal hemorrhage ^17^. Numerous modifications have been explored over the years to reduce the rate of these. They include a smaller scleral flap ^18^, limbus-versus fornix-based conjunctival closure ^19^, releasable flap sutures ^20, 21^, a combination of trabeculectomy with deep sclerectomy ^22^, different concentrations and exposure times of MMC ^23^ or sutureless tunnel trabeculectomy without iridectomy ^24^. The iridectomy, which is part of traditional trabeculectomy with a trabecular block excision, can cause hyphema, inflammation, posterior synechiae, iridodialysis, and cataracts ^25, 26^. FTO addresses some of these issues by creating a more spread-out intake of aqueous humor, thereby reducing iris aspiration and avoiding the need for an iridectomy. The trabeculotomy and sclerectomy ^8, 27, 28^, that are part of the FTO, were meant to remove some of the post-trabecular outflow resistance ^29– 31^.

We used matching, a nonparametric method of controlling the confounding influence of pretreatment variables in observational data ^32^. Before matching, FTO and TE had significant differences. We have previously used coarsened exact matching, propensity score matching, and, more recently, exact matching. Coarsened exact matching applies multiple imputations to fill in missing data to not distort any relationships contained in the data while enabling the inclusion of all observed data from moderately uneven groups. Such was the case when we compared patients with a primary IOP indication to patients with a mixed indication for both cataract removal and IOP reduction ^33– 35^. Propensity score matching is helpful to compare even more divergent groups, for instance, patients undergoing tube shunt surgery with patients undergoing trabectome surgery ^36, 37^. By contrast, exact matching is well suited to compare similar pathological conditions and similar treatments, for instance, phaco-iStent or phaco-trabectome ^38, 39^. A downside of exact matching is that a certain number of datasets must be excluded from the analysis because the algorithm accepts only identical primary criteria matches. Overall, our data loss was acceptable because of the high similarity that already existed at baseline. We were able to retain a large number of eyes (about 45%) as identical pairs of preoperative IOP and medication to focus on the preeminent questions of success in IOP and medication reduction. We found that FTO was as successful as TE with a similar reduction of IOP and medications. Both had a similar intervention and complication rate, notwithstanding numerical hypotony within the first six weeks after surgery. We observed a remarkably low rate of hyphema compared to *ab interno* trabeculectomy ^40^ that occurs when the IOP is at or below episcleral venous pressure allowing blood to reflux into the anterior chamber. This could indicate reduced patency of collector channels in advanced glaucoma that qualifies for filtering surgery, which has been *observed ex vivo* ^41^.

Trabeculotomy *ab externo* has been applied to adult POAG before but, compared to TE, was noted to have a lower success rate of 70% at one year, presumably due to a reapproximation or regeneration of the disrupted trabecular meshwork ^42^. A study by Chihara *et al.* was in agreement with this, finding that a modified trabeculotomy *ab externo* lowers IOP to an average near 16 mmHg in a safe fashion ^43^, by not as much as TEs. Ogawa *et al.* compared a nonpenetrating trabeculectomy with or without trabeculotomy ^44^ using a technique that was very similar to the one applied in our study, except without MMC. Despite the absence of this antifibrotic, the authors achieved a two-year IOP of 13 mmHg, not unlike our patients. 

Our two-year TE results match Kirwan *et al.*’s multicenter study of TE with an iridectomy in 428 eyes well ^45^. These eyes achieved an IOP of 12.4±4 mmHg and 80% did not have to use glaucoma medications anymore, slightly more than in our study population. The authors performed needling in 17% of patients, not unlike our rates, and numerical hypotony during the first six months occurred in 7.2% of patients, which is in a similar range as our 9% in FTO and 2% in TE. Leakage was observed in 14% compared to 9% in our FTO and 2% in our TE. However, Kirwan *et al.*’s cataract surgery rate was 31%, much higher than our 1% in both FTO and TE, which might represent a difference in practice pattern or simply easier access to this elective procedure, which is done as an outpatient surgery in the UK.

It is interesting to note that TEs in the Tube Versus Trabeculectomy (TVT) study were performed without an iridectomy ^46^. In that study, IOP reduced from 25.6±5.3 mm Hg to 12.7±5.8 mm Hg at one year while the number of glaucoma medications declined from 3.0±1.2 to 0.5±0.9 ^47^, which is also relatively similar to our results although these investigators included eyes with prior intraocular surgery, including glaucoma procedures. An early complication rate of 37% was observed by these authors, primarily consisting of a shallow or flat anterior chamber in 20% and choroidal effusions in 10% ^6^. In another study of TE without an iridectomy by Jea *et al.*, IOP reduced from 26.3±10.9 mmHg to 10.2±4.1 mmHg at two years ^48^. The number of glaucoma medications decreased from 2.2±1.6 to 0.5±1.0. A complete success occurred in 76.6% at one year and 66.2% at two years, matching ours.

In all of these studies, TE was performed as an outpatient patient procedure, a practice that emerged in the late 1980s ^49, 50^ and became a standard for most types of eye surgeries and countries ^51– 54^. Even before the now-common use of antifibrotics, there was no significant difference in success or complication rates between inpatients and outpatients ^50^, an observation that has been confirmed with antifibrotics as well ^53^. In the country of our study, TE and FTO are only reimbursable as inpatient procedures. It has been argued that meticulous micromanagement after TE for several days may be associated with better long-term outcomes of TE. However, this hypothesis is challenged by the present study and by the findings of others ^49, 50, 53, 55^. One could argue that the considerably lower cataract surgery rate in our data compared to Kirwan *et al.* ^45^ might indicate a higher threshold for cataract surgery. These would have typically also been done as inpatient procedures to better handle post-cataract surgery bleb care.

Our study was limited by its retrospective nature and nonrandomized design. In patients with surgery in both eyes, we had selected the first eye undergoing glaucoma surgery for our analysis to take advantage of a longer history with more data. This selection might have favored the eye with more severe glaucoma. However, as this occurred in both groups, a similar bias will have occurred. Certainly, randomized controlled trials are a more sophisticated tool to reduce bias when trying to detect differences. However, given that exact matching already allows for a highly balanced comparison of retrospective data, such an effort might be difficult to justify. The indication for postoperative interventions and length of hospitalization was at the discretion of the treating physicians and was not standardized for both groups. Despite reducing confounding through the exact matching of IOP and medications, other confounding factors might have contributed to small differences in early postoperative patient management, as reflected by the fact that FTO patients received more 5-FU injections. Although these patients had a higher rate of numerical hypotony they were hospitalized for a slightly shorter time and experienced results that were not significantly different.

In conclusion, our results are largely in line with other FTO and TE outpatient studies. Combining elements from both yields reasonable two-year rates of surgical success, postoperative complications, and interventions while avoiding an iridectomy.

## Data availability

### Underlying data

Open Science Framework: Retrospective evaluation of 2-year results with a filtering trabeculotomy in comparison to conventional trabeculectomy by exact matching, https://doi.org/10.17605/OSF.IO/KDYF3 ^56^.

Data are available under the terms of the Creative Commons Zero “No rights reserved” data waiver (CC0 1.0 Public domain dedication).
