# Supplementary material for: Retrospective evaluation of two-year results with a filtering trabeculotomy in comparison to conventional trabeculectomy by exact matching
Source: F1000Res. 2021 Jun 4;9:1245. Originally published 2020 Oct 15. [Version 2] doi: 10.12688/f1000research.26772.2 (PMC7676392; doi:10.12688/f1000research.26772.2)
Supplement: Peer review history

# Peer review: Reviewer response for version 1

**DOI:** 10.5256/f1000research.29560.r73660

**Published:** 2020-12-08

**Authors:** Christian van Oterendorp

**Affiliations:** 1 Department of Ophthalmology, University Medical Center Göttingen, Göttingen, Germany

The authors present a highly controlled retrospective study comparing two glaucoma filtration surgery techniques. The statistical methods applied are sound, the presentation is well structured and transparent, the discussion is well balanced and covers the current literature.

 A few more points may be discussed:

 The choice of the exact matching criteria. Obviously, extending the matching criteria reduces the number of available matches. But besides the criteria chosen the authors may discuss whether the inclusion of criteria directly linked to failure, such as the presence of drug related ocular surface inflammation, could have led to a further reduction of a significant confounding factor. More precisely, if a grading system for preoperative ocular surface inflammation existed, this could have been included as a matching criterion. However, given the retrospective nature of this study, such a grading might not have been available.

 Discussion. The results suggest that both interventions may be used interchangeably. Could the authors provide arguments which speak in favour or against one or the other technique? - maybe with respect to certain details, such as:

1. Lens status. Should FTO be considered rather in phakic eyes as the risk for cataract development may be lower? But why was the rate of pseudophakic patients significantly higher in FTO compared to TE?
2. Surgery time and/or need for general anaesthesia (TE faster? therefore better for local anaesthesia?).

Is the work clearly and accurately presented and does it cite the current literature?

Yes

If applicable, is the statistical analysis and its interpretation appropriate?

Yes

Are all the source data underlying the results available to ensure full reproducibility?

Partly

Is the study design appropriate and is the work technically sound?

Yes

Are the conclusions drawn adequately supported by the results?

Yes

Are sufficient details of methods and analysis provided to allow replication by others?

Yes

Reviewer Expertise:

Glaucoma

I confirm that I have read this submission and believe that I have an appropriate level of expertise to confirm that it is of an acceptable scientific standard.

# Peer review: Reviewer response for version 1

**DOI:** 10.5256/f1000research.29560.r74186

**Published:** 2020-11-18

**Authors:** Igor I. Bussel

**Affiliations:** 1 Gavin Herbert Eye Institute, University of California, Irvine School of Medicine, Irvine, CA, USA

Most glaucoma surgery clinical trials are often non-randomized, usually limited by a short duration of follow-up (<1 year), lack hypothesis, and appropriate comparison to a gold-standard. The investigators of this trial managed to overcome lack of randomization by using novel exact matching methodology. This allowed for a balanced comparison of retrospective data. FTO was compared to the gold standard, TE. It is unclear if the exact matching process created a sample bias of cases with higher success. The authors demonstrated that FTO and TE yield similar two-year rates of surgical success, postoperative complications, and interventions.

Is the work clearly and accurately presented and does it cite the current literature?

Yes

If applicable, is the statistical analysis and its interpretation appropriate?

Yes

Are all the source data underlying the results available to ensure full reproducibility?

No

Is the study design appropriate and is the work technically sound?

Yes

Are the conclusions drawn adequately supported by the results?

Yes

Are sufficient details of methods and analysis provided to allow replication by others?

Yes

Reviewer Expertise:

Glaucoma Clinical Trials, Glaucoma Surgery Outcomes, Glaucoma Imaging, Glaucoma/Cataract Surgery

I confirm that I have read this submission and believe that I have an appropriate level of expertise to confirm that it is of an acceptable scientific standard.

# Peer review: Reviewer response for version 1

**DOI:** 10.5256/f1000research.29560.r73193

**Published:** 2020-10-30

**Authors:** Marc Töteberg-Harms

**Affiliations:** 1 Department of Ophthalmology, University Hospital Zurich, Zurich, Switzerland

The authors present a retrospective study comparing two surgical interventions for the management of glaucoma. The use of exact matching is a major advantage of their study design.

 It is unclear if only one eye per patient was included and, if not, if a statistical model to account for two-eye-effect was used.

 A post-hoc power analysis could further improve this excellent paper to prove that the sample size of 44 eyes per group was sufficient to find a difference or no difference between the two study arms

Is the work clearly and accurately presented and does it cite the current literature?

Yes

If applicable, is the statistical analysis and its interpretation appropriate?

Partly

Are all the source data underlying the results available to ensure full reproducibility?

No

Is the study design appropriate and is the work technically sound?

Yes

Are the conclusions drawn adequately supported by the results?

Yes

Are sufficient details of methods and analysis provided to allow replication by others?

Yes

Reviewer Expertise:

Glaucoma, cataract.

I confirm that I have read this submission and believe that I have an appropriate level of expertise to confirm that it is of an acceptable scientific standard.

# Response

**Published:** 2020-11-10

**Authors:** Nils Loewen

**Affiliations:** School of Medicine, University of Wurzburg, Germany

Manuscript No. 9:1245 ( https://doi.org/10.12688/f1000research.26772.1)

**Title: Retrospective evaluation of two-year results with a filtering trabeculotomy in comparison to conventional trabeculectomy by exact matching**

 Alicja Strzalkowska, Piotr Strzalkowski, Yousef Al Yousef, Jost Hillenkamp, Franz Grehn, Nils A. Loewen

**Authors:** We appreciate the helpful comments and have provided our replies below. We trust that we could address the concerns that were raised. 

**Marc Töteberg-Harms:** The authors present a retrospective study comparing two surgical interventions for the management of glaucoma. The use of exact matching is a major advantage of their study design. It is unclear if only one eye per patient was included and, if not, if a statistical model to account for two-eye-effect was used.

**Authors:** Thank you for pointing this out. We have updated the Method to clarify that only one eye was included per patient: “Only one eye was included per patient. If two eyes had been operated on, the first eye was chosen to take advantage of a longer history.” 

 We added to Discussion: “In patients with surgery in both eyes, we had selected the first eye undergoing glaucoma surgery for our analysis to take advantage of a longer history with more data. This selection might have favored the eye with more severe glaucoma. However, as this occurred in both groups, a similar bias will have occurred.” 

**Marc Töteberg-Harms:** A post-hoc power analysis is missing to prove that the sample size of 44 eyes per group is sufficient to prove a difference or no difference between the two study arms.

**Authors:** Thank you for raising this issue. We had previously written in the second last paragraph of the Discussion “We determined that we had a testing power above 80% to detect a difference of more than 2 mmHg 1 year (alpha= 0.05), yet but no significant difference was found.” We have now deleted this statement and inserted the text as described below. Our power study before commencing the study indicated that we would need 39 eyes per group to detect a difference of at least 20%. Our posthoc calculation showed that we had a power to detect a difference of 16% at 1 year and 17.6% at 2 years. We added to Methods: “Our power calculation before commencing the study indicated that we would need 39 eyes per group to detect a difference of at least 20% at a power of 80% and alpha of 0.05 (continuous endpoints, two independent sample study). We then performed a post-hoc analysis for IOP at year 1 and 2 and determined the effect size using Cohen’s d (d (.01) = very small, d (.2) = small, d (.5) = medium, d (.8) = large, d (1.2) = very large, and d (2.0) = huge [1]).”

 We added to Results: “The posthoc analysis showed that we could have detected an IOP difference of 16.1% at one year and an IOP difference of 17.6% at two years with a power above 80%. Cohen’s d was 0.32 at one year and 0.29 at two years, respectively, in both cases classifying as small.”

**Marc Töteberg-Harms:** *Are all the source data underlying the results available to ensure full reproducibility?* - No

**Authors:** We have made all underlying data available at Open Science Framework: Retrospective evaluation of 2-year results with a filtering trabeculotomy in comparison to conventional trabeculectomy by exact matching, https://doi.org/10.17605/OSF.IO/KDYF356 . Data are available under the terms of the Creative Commons Zero “No rights reserved” data waiver (CC0 1.0 Public domain dedication).

**References**

 1. Sawilowsky SS (2009) New Effect Size Rules of Thumb. J Mod Appl Stat Methods 8:26
